# Fatty acid binding protein 5 promotes the proliferation, migration, and invasion of hepatocellular carcinoma cells by degradation of Krüppel-like factor 9 mediated by miR-889-5p via cAMP-response element binding protein

**DOI:** 10.1080/15384047.2022.2094670

**Published:** 2022-07-11

**Authors:** Yanping Tang, Kezhi Li, Bangli Hu, Zhengmin Cai, Jilin Li, Hao Tao, Ji Cao

**Affiliations:** a Department of Research, Guangxi Medical University Cancer Hospital, Nanning, Guangxi, China; b Key Laboratory of Early Prevention and Treatment for Regional High Frequency Tumor (Guangxi Medical University), Ministry of Education, Nanning, Guangxi, China

**Keywords:** Fatty acid binding protein 5, cAMP-response element binding protein, miR-889-5p, Krüppel-like factor 9, hepatocellular carcinoma

## Abstract

Mounting evidence has demonstrated that fatty acid binding protein 5 (FABP5) is commonly upregulated in many human malignancies. However, the mechanisms explaining the involvement of FABP5 in hepatocellular carcinoma (HCC) remain unclear. In this study, we demonstrated the involvement of FABP5 and its downstream signaling molecules in HCC progression. We first confirmed that FABP5 expression was upregulated in HCC. Additionally, FABP5 promoted HCC cells proliferation, migration, and invasion. Mechanistic investigation showed that FABP5 could improve cAMP-response element binding protein (CREB) phosphorylation. Meanwhile, CREB, as a transcription factor, upregulated the miR-889-5p expression by binding to the miR-889-5p promoter region. Consequently, miR-889-5p led to downregulation of Krüppel-like factor 9 (KLF9) by binding to the 3ʹ-UTR of the KLF9 mRNA, potentiating the PI3K/AKT signaling pathway and promoting the proliferation, migration, and invasion of HCC cells. Our findings have identified a FABP5/CREB/miR-889-5p/KLF9 axis for HCC progression, and we postulate that blocking this key signaling pathway may represent a promising strategy for HCC treatment.

## Introduction

The occurrence and development of liver cancer is a complex process involving multi-factor, multi-stage, and multi-gene mutation accumulation. It is of utmost importance in the prevention and treatment of liver cancer to identify certain key genes in liver cancer. Currently, a critical challenge is distinguishing key molecules that confer a selective advantage to tumorigenesis from the random alterations due to the inherent genomic instability, gene linkage, and spontaneous mutagenesis.^1,2^ In our previous study, we found that some genes in the differentially expressed gene profile of human HCC were shared by HCC tissues from other animals with liver cancer.^3^ Therefore, we speculate that it is possible to identify key genes that affect the development of liver cancer by exploring common gene changes between human HCC and other species. Hence, we carried out a strategy of cross-species comparative oncogenomics by collecting the differentially expressed genes in the liver cancer tissues of tree shrews (Tupaia belangeri) and the differentially expressed proteins in the liver cancer tissues of rats, combined with the Gene Expression Omnibus (GEO) database (including humans and mice).^4,5^ Among the different potential targets, the epidermal fatty acid binding protein 5 (FABP5), also known as E-FABP, attracted our attention.

FABP5, a member of the fatty acid binding protein family, is a highly conserved small molecule protein derived from epidermal cells and is located in the cytoplasm.^6^ As a carrier of hydrophobic ligands (including retinoic acids and various long-chain fatty acids), it not only participates in the binding, transport, storage, and metabolism of long-chain fatty acids, which provide energy and raw materials for cell growth but also affects signal transduction in cell growth.^7,8^ Thus far, FABP5 has been confirmed as an oncogene in prostate cancer, gliomas, breast cancer, colorectal cancer, and so on.^9–12^

Mounting evidence has recently revealed the molecular mechanism by which FABP5 promotes tumor development. Researches have shown that FABP5 may be involved in tumorigenesis by influencing lipid metabolism, including promoting lipid droplet decomposition, enhancing de novo fatty acid synthesis pathways, and upregulating the expression of key enzymes involved in lipid metabolism and lipolysis.^13,14^ FABP5 enhances lipid reprogramming by promoting hypoxia-inducible factor (HIF-1) synthesis, preventing the interaction of factor-inhibiting hypoxia-inducible factor (FIH)/HIF-1, and improving HIF-1 activity in liver cancer cells.^15^ Additionally, FABP5 can regulate tumor progression by activating key signaling pathways. In prostate cancer, FABP5 activates the NF-κB signaling pathway by increasing reactive oxygen species (ROS) and protein kinase C production and induces inflammation and the production of IL-6 and IL-8.^16^ FABP5 can promote angiogenesis in HCC by activating the IL6/STAT3/VEGFA pathway.^17^ However, the specific molecular mechanism by which FABP5 regulates the proliferation and migration of hepatoma cells has not been clearly defined, and it needs to be further elucidated. Therefore, this study aimed to investigate the role and potential molecular mechanisms of FABP5 in HCC.

## Materials and methods

### Patients and tissue samples

48 HCC tissues and corresponding adjacent normal tissues from Guangxi Medical University Cancer Hospital between January 2018 and February 2019 were collected. All HCC patients, who received no pre-operative radiotherapy, chemotherapy and surgical treatment, excluding other tumors and serious psychosomatic diseases, were initially diagnosed as hepatocellular carcinoma by pathology after operation. Tumor staging was classified into stages A, B, C, and D following the Barcelona Clinic Liver Cancer classification criteria.^18^ This study was approved by the Ethical Committee of Guangxi Medical University Cancer Hospital (No. LW2021086). All participants signed a written informed consent.

### Cell culture

In the previous experiment, the expression of FABP5 mRNA was verified in four kinds of HCC cells, including HepG2, Huh7, SK-Hep-1, Hep3B cells. It was found that the mRNA expression level of FABP5 was higher in HepG2 and SK-Hep-1, which was used for this study. The HCC cell lines HepG2 and SK-Hep-1 were purchased from the Chinese Academy of Sciences Cell Bank (Shanghai, China). Cells were cultured in Dulbecco’s Modified Eagle Medium (DMEM) medium containing 10% fetal bovine serum (FBS) and 1% antibiotics (100 U/ml penicillin and 100 mg/ml streptomycin) and incubated at 37°C with 5% CO_2。_

### Lentiviral transfection

FABP5-knockdown, FABP5-overexpressed, and Krüppel-like factor 9 (KLF9)-knockdown and their corresponding control lentiviruses were constructed by Hanyin Biotechnology Limited Company (Shanghai, China). The shRNA target sequences are listed in Supplementary Table 1. HCC cells were seeded into 6-well plates, and 10 μL of the lentivirus with polybrene (with a final concentration of 5 g/ml) were added per well. The infected cells were selected by Puromycin (2 μg/ml) after 48 h of transduction.

### SiRNA and vector infection

miR-889-5p mimic, siRNAs of NF-κb, c-Myc, cAMP-response element binding protein (CREB), and the corresponding negative controls were synthesized by RiboBio (Guangzhou, China). These were transfected into HCC cells using Lipofectamine 3000 (Invitrogen, Cat. no. L3000015) according to the manufacturer’s protocol. miR-889-5p mimic and the siRNAs sequences are listed in Supplementary Table 2.

### RT-qPCR

Total RNA extracted by TRIzol reagent (Invitrogen, Cat. no. 15596026) was reversely transcribed into cDNA using a PrimeScript™ RT Reagent kit (Takara, Cat. no. RR037A) according to the manufacturer’s guidelines. qPCR was performed using the SYBR® Premix Ex Taq kit (Takara, Cat. no. RR820A). GAPDH and U6 were used as the reference genes for mRNA and miRNA respectively. The results were analyzed using the 2-^ΔΔCT^ method.^19^ Primer sequences used in this study are listed in Supplementary Table 3.

### Western blot analysis

The total protein was extracted from the prepared cells and tissues by radioimmunoprecipitation assay (RIPA) lysis buffer containing 1% phenylmethylsulfonyl fluoride (1 mM), followed by the quantification. 50 μg of the assigned protein was separated using 10% sodium dodecyl sulfate-polyacrylamide gel electrophoresis and loaded on polyvinylidene fluoride membranes. Nonspecific antigens were blocked in 5% skimmed milk for 1 h. Subsequently, the membranes were incubated overnight at 4°C with the different primary antibodies as follows: anti-FABP5 rabbit polyclonal antibody (Abcam, Cat. no. ab84028), anti-KLF9 rabbit polyclonal antibody (Abcepta, Cat. no. Ap16249a), anti-PI3K rabbit polyclonal antibody (Cell Signaling Technology, Cat. no. 3811), anti-Phospho-PI3K rabbit polyclonal antibody (Cell Signaling Technology, Cat. no. 13857), anti-AKT rabbit monoclonal antibody (Cell Signaling Technology, Cat. no. 4685), anti-Phospho-AKT rabbit monoclonal antibody (Abways, Cat. no. cy6569), anti-mTOR rabbit monoclonal antibody (Cell Signaling Technology, Cat. no. 2986), anti-Phospho-mTOR rabbit monoclonal antibody (Cell Signaling Technology, Cat. no. 5536), anti-CREB rabbit monoclonal antibody (Cell Signaling Technology, Cat. no. 9197), anti-Phospho-CREB rabbit monoclonal antibody (Cell Signaling Technology, Cat. no. 9198), anti-β-actin mouse monoclonal antibody (Santa Cruz, Dallas, Cat. no. sc-8432), and anti-GAPDH mouse monoclonal antibody (Abcam, Cat. no. ab59164).

### Cell viability

The transfected cells were harvested after transfection for 48 hours and seeded into a 96-well plate. At 0, 24, 48, 72, and 96 h after cell attachment, 10% Cell Counting Kit (CCK)-8 solution (Dojindo, Cat. no. CK04) was added. Cells were incubated at 37°C with 5% CO_2_ for 1 h. Absorbance was measured at 450 nm using a microplate reader (Berthold).

### Transwell assay

Transwell assay was performed to detect cell migration and invasive ability. For the invasion assay, the transwell chamber was coated with Matrigel® (BD Biosciences, Cat. no. 354480). 10^5^ cells were added to the upper chamber, followed by incubation for 24 h. The migratory cells on the lower surface of the insert were fixed in 4% paraformaldehyde and methanol, and stained with crystal violet.

### Cell cycle assay

10^6^ cells for each group were harvested and washed twice with cold phosphate-buffered saline (PBS). Then, the cells were fixed with 70% ethanol overnight and stained with propidium iodide (PI; Multi Science, Cat. no. 70-AP105-60) solution containing RNase A (10 μg/ml) for 30 min. The cells were analyzed on a FACSCalibur™ flow cytometer (BD Biosciences).

### Apoptosis assay

The collected cells were stained with 20 μl of PI and 20 μl of fluorescein isothiocyanate (FITC; Multi Science, Cat. no. 70-CCS012) for 20 min at room temperature. Apoptotic cells were analyzed on a FACSCalibur™ flow cytometer (BD Biosciences).

### Dual-luciferase assay

The full-length KLF9 UTR (wild-type or mutant sequence) and miR-889-5p promoter (wild-type or mutant sequence) were cloned into the psiCHECK-2 vector (GenePharma). 293 T cells were then co-transfected with the miR-889-5p mimic and wild-type KLF9 or mutant KLF9 luciferase reporter plasmids to study the interaction between miR-889-5p and KLF9. They were also co-transfected with the CREB overexpression plasmid and wild-type miR-889-5p promoter or mutant miR-889-5p promoter to study the interaction between CREB and miR-889-5p. After 48 h of incubation, the collected cells were lysed. Luciferase activity was detected using a dual-luciferase assay kit (Promega, Cat. no. E1910) according to the manufacturer’s protocol.

### Xenograft tumor growth model

The Ethical Committee of Guangxi Medical University Cancer Hospital approved the animal experiments (No. LW2021087). All experimental procedures and animal care were conducted in accordance with institutional ethics guidelines. A total of 12 BABL/c nude mice, 5 weeks old, weighing 18–20 g, were randomly divided into sh-negative control (NC) group (transfected cells with negative control empty lentiviral particles), and shFABP5 group (transfected cells with lentiviruses carrying shRNA to inhibit FABP5). Approximately 1 × 10^6^ SK-Hep-1 cells were injected into the right axilla (0.5 cm) of the mice. The longest and shortest diameter of the tumor were measured and the tumor volume was calculated every week. Four weeks after injection, the nude mice were euthanized, and the xenograft tumors were harvested and weighed.

### Immunohistochemistry (IHC) assay

Xenograft tumors were fixed in 4% paraformaldehyde for 48 h. The fixed tumors were then dehydrated, embedded in paraffin, and cut into 5 μm sections. The sections were incubated at 4°C overnight with rabbit anti-human FABP5 primary antibody (Proteintech, cat. no. 12348-1-AP) and then with goat anti-rabbit secondary antibody (Proteintech, cat. no. PR30009) for 50 min at room temperature. Ten random fields of positive cells were examined at 200X magnification.

### Statistical analysis

Statistical analyses were performed using the SPSS software (v19.0, IBM Inc., Armonk, NY, USA). Continuous variables were presented as mean ± standard deviation. The differences between two groups were verified using the Student’s t-test. The Kruskal-Wallis test and Mann-Whitney U test were performed for group comparisons. Categorical variables were compared using the χ2 test or Fisher’s exact test. Univariate survival analysis was performed using the Kaplan-Meier method. Correlational analysis was performed using Pearson’s correlation coefficient. In this study, p < .05 was defined as a significant difference.

## Results

### FABP5 was upregulated in HCC samples

**Figure 1.**
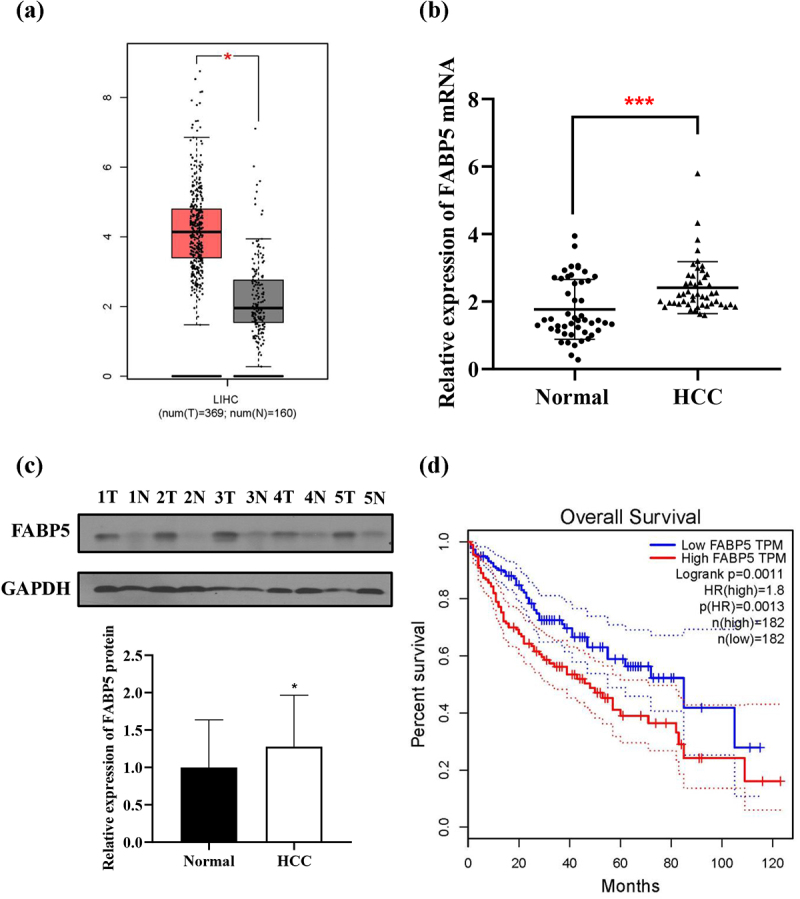
FABP5 expression was upregulated in HCC. a, expression of FABP5 in HCC samples (N = 369) and normal samples (N = 160) from the TCGA data set. b, relative expression of FABP5 mRNA in HCC tissues compared with adjacent normal tissues were detected by RT-qPCR. c, relative expression of FABP5 protein in HCC tissues and adjacent normal tissues were detected by western blotting. Representative images of FABP5 expression in 48 paired HCC tissues(t) and adjacent normal tissues (n). d, the expression of FABP5 was negatively correlated with overall survival time in HCC patients from the TCGA data set. *P < .05 and ***P < .001 as compared with the vehicle control.

FABP5 expression in HCC tissues was significantly higher than that in adjacent normal tissues from the TCGA data set using GEPIA database (http://gepia.cancer-pku.cn) (Figures 1a). We examined FABP5 expression in 48 paired HCC tissues and adjacent normal tissues using qPCR and consistently found that it was significantly upregulated in the tumor tissues (Figure 1b). The results were further confirmed by western blotting (Figures 1c). The median level of FABP5 protein in the 48 HCC tissues was calculated and divided into high (n = 24) and low (n = 24) FABP5 expression groups. By analyzing the clinicopathological characteristics and clinical outcomes of the 48 HCC patients, we discovered that the expression of FABP5 was closely related to tumor staging and postoperative metastasis (Table 1, p < .05). In addition, Kaplan-Meier curves demonstrated a poor prognosis in HCC patients in the high FABP5 expression group (from TCGA data set) (Figure 1d). Taken together, FABP5 is a critical gene involved in the occurrence and development and of HCC.

**Table 1.** Correlation between FABP5 expression and clinicopathological characteristics

		FABP5			
Parameters	Number of Cases	Low Expression	High Expression	χ2	P -value
**Age (years)**				0.375	0.540
<60	32	17	15		
≥60	16	7	9		
**Gender**				0.949	0.330
Male	35	19	16		
Female	13	5	8		
**Histological grade**				0.087	0.768
Low	29	15	14		
High	19	9	10		
**Tumor size(cm)**				2.254	0.133
≤5	12	8	4		
>5	36	15	21		
**TNM**				6.762	0.009*
A-B	25	17	8		
C-D	23	7	16		
**Lymph node metastasis**				6.206	0.013*
Yes	15	3	12		
No	33	21	12		

Note: *P < 0.05 or was considered significant (Chi-square test between two groups).

### Knockdown of FABP5 suppresses proliferation, migration, and invasion of HCC

**Figure 2.**
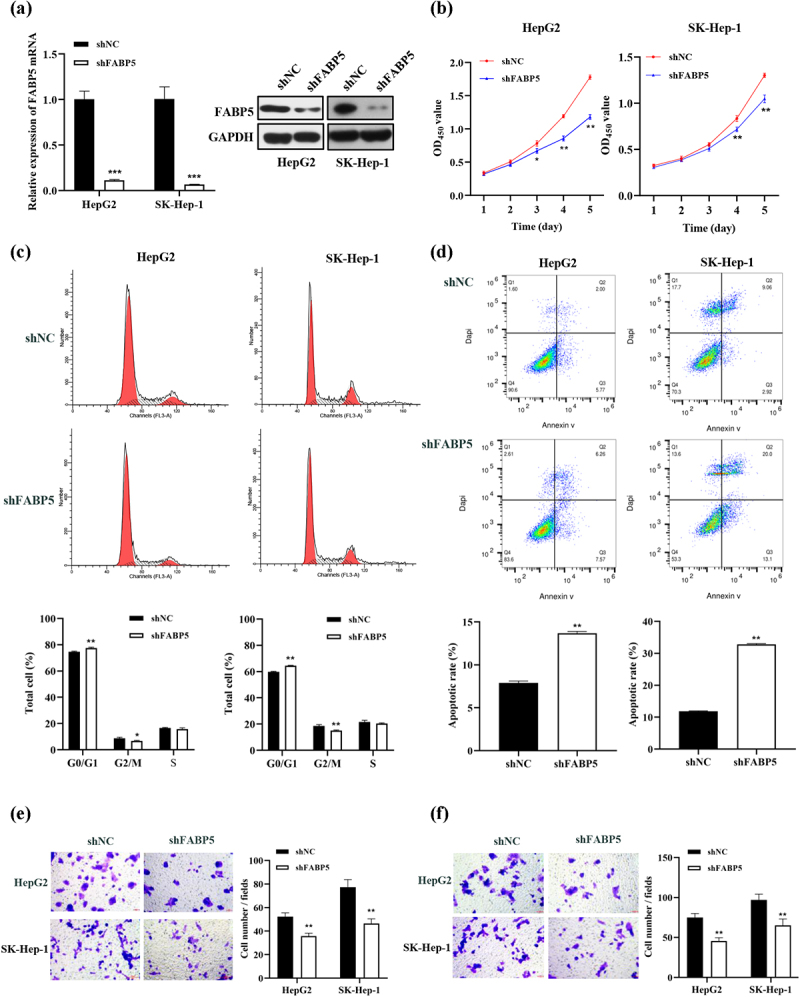
FABP5 knockdown inhibited cell growth, migration and invasion in HCC cells. a, the mRNA and protein expression levels of FABP5 were analyzed in FABP5-knockdown HepG2 and SK-Hep-1 cells by RT-qPCR and western blotting. b, the growth curve of HCC cells was determined by CCK-8 assay. c, PI fluorescence pattern was applied for cell-cycle distribution in HCC cells transfected with shFABP5 or shNC. d, HCC cells transfected with shFABP5 or shNC were stained with Annexin-V FITC and PI to be measured apoptosis. Apoptotic cells are presented in the right-lower (Q3, early apoptosis) and right-upper (Q2, late apoptosis) quadrants of the plots. e, transwell assay revealed the migration abilities of HCC cells transfected with shFABP5 or shNC. f, transwell assay revealed the invasive abilities of HCC cells transfected with shFABP5 or shNC. Results are presented as mean ± S.E.M. (N = 3). Results were averaged from three independent experiments and presented as percentage of control levels. *p < .05, **p < .01 and ***p < .001 as compared with the vehicle control. FITC, fluorescein isothiocyanate; PI, propidium iodide.

To investigate the role of FABP5 in HCC progression, HepG2 and SK-Hep-1 cells were transfected with FABP5 shRNA, and FABP5 expression was verified. These cells were successfully transfected with FABP5 shRNA, as indicated by the significantly lower levels of FABP5 mRNA in the shFABP5 group compared with their respective negative controls (P < .001). Western blot analysis revealed that FABP5 protein expression dramatically decreased in HepG2 and SK-Hep-1 cells after transfection (Figure 2a). As demonstrated in Figure 2b, the viability of these cell lines was significantly reduced in the shFABP5 group when compared with the shNC group on days 4 and 5 following transfection (HepG2 cells at days 3, 4, and 5 following transfection). To further explore the mechanisms underlying the effects of FABP5 on HCC cell proliferation, flow cytometry was used to analyze cell cycle distribution and apoptosis in FABP5 knockdown and NC groups. As shown in Figure 2c, the cell population in the G0/G1 phase increased compared to that in the NC group after transfection with FABP5 shRNA. Additionally, the G2/M phase ratio of the shFABP5 cells was lower than that in the shNC group. This indicated that FABP5 silencing led to cell cycle arrest at the G0/G1 phase in HepG2 and SK-Hep-1 cells. Next, we explored the effects of FABP5 on apoptosis. Compared with the control cells, the FABP5 knockdown group showed a significantly increased apoptotic proportion, indicating that FABP5 knockdown can induce apoptosis in HCC cells (Figures 2d). These data indicated that FABP5 increased the proliferation of tumor cells by promoting cell division and inhibiting apoptosis. Transwell assays were used to investigate the effects of FABP5 on the migration and invasion of HCC cells. As indicated in Figures 2(e,f), our results showed that the migration and invasion of HepG2 and SK-Hep-1 cells with FABP5 knockdown was significantly lower than that of the vector-transfected control group. FABP5 silencing significantly inhibited the migration and invasive abilities of HCC cells. These data confirmed that the FABP5 knockdown significantly inhibited the proliferation, migration, and invasion of HepG2 and SK-Hep-1 cells.

### Suppression of FABP5 inhibits tumor growth in vivo

**Figure 3.**
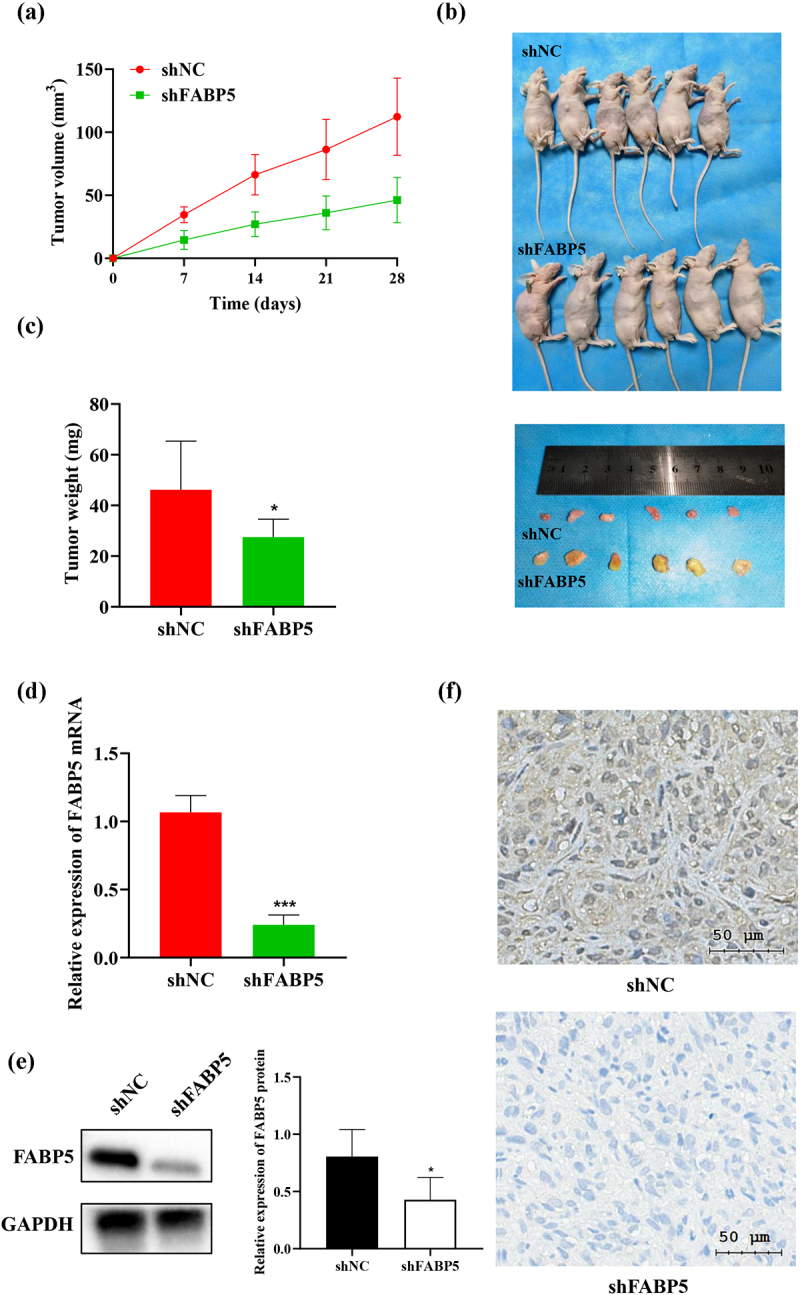
Decreased expression of FABP5 inhibited the growth of HCC cells in vivo. a, HepG2 cells and HepG2 cells stably transfected with shFABP5 RNA or shNC were implanted into nude mice by subcutaneous injection. Tumor volume was measured each 7 days by a calliper until the end of the experiment. The tumor growth curve was drawn. b, the mice and the tumors of different groups on the 28th day after injection. c, the weight of each xenograft tumor was measured at the end of the experiment. d, the differences in FABP5 mRNA levels between the shFABP5 group and the control groups were detected by RT-qPCR. e, the protein levels of FABP5 in the shFABP5 group and the control groups were measured by western blotting. f, the tumors were analyzed by immunohistochemical staining with anti-FABP5. Original magnification: 400 × . ***p < .001 as compared with the vehicle control.

SK-Hep-1 cells successfully transfected with stable shFABP5 RNA were injected into the BALB/c mice to create a xenograft tumor model. Mice were assigned to the shFABP5 and shNC groups. We found that tumors grew more rapidly in the shNC groups, as shown in Figure 3a. The mice were sacrificed on the 28th day after injection. Tumors from the shNC group were larger and heavier than in the FABP5 shRNA group, as shown in Figures 3(b,c). We performed RT-qPCR, IHC staining, and western blot analysis to evaluate FABP5 expression at the transcriptional and translational levels. Compared to the shNC groups, FABP5 mRNA and protein expression in the FABP5 shRNA group was significantly decreased (P < .001) Figures 3(D-e). Representative images of the immunohistochemical staining in shNC and shFABP5 groups were shown in figure 3f.

### The KLF9 gene was the downstream target of FABP5

**Figure 4.**
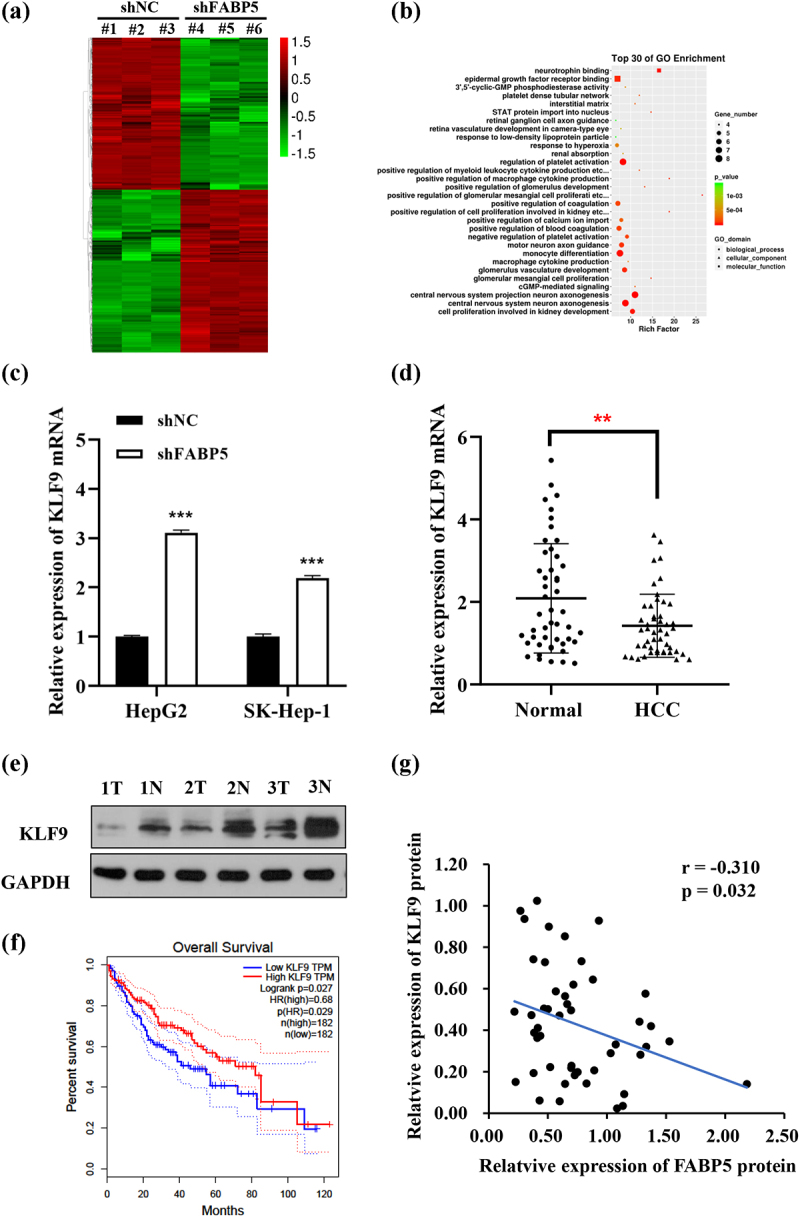
The expression of KLF9 was negatively correlated with FABP5. a, gene microarray analysis were applied to examine the differential expression genes in SK-Hep-1 cells before and after FABP5 knockdown (FC > |2|; P < .01). b, gene ontology (GO) enrichment analysis of differential expression genes in accordance with the biological processes, cellular component, and molecular function. c, KLF9 mRNA expression in HepG2 and SK-Hep-1 cells with FABP5 knockdown determined by RT-qPCR. d, the KLF9 expression in HCC tissue and adjacent tissues were detected by RT-qPCR. e, relative expression of KLF9 protein in HCC tissues and adjacent normal tissues were detected by Western blotting. Representative images of KLF9 expression in 48 paired HCC tissues (t) and adjacent normal tissues (n). e, the expression of KLF9 was positively correlated with overall survival time in HCC patients from the TCGA data set. F, A negative correlation was found between the protein expression level of FABP5 and KLF9 in HCC samples. *p < .05, **p < .01, and ***p < .001 as compared with the vehicle control.

In order to better understand the molecular mechanisms by which FABP5 regulates the progression of HCC, we analyzed potential downstream target genes of FABP5 using transcriptional profiling of FABP5 knockdown SK-Hep-1 cells compared with the control group. A total of 832 genes were identified as having significant changes (FC > |2|; P < .01). Among these, 432 genes were upregulated, and 400 genes were downregulated in the SK-Hep-1 cells following FABP5 knockdown. An interactive heat map shows the abundance of these differentially expressed (DE) genes (Figure 4a). According to Gene Ontology (GO) analysis, the DE genes were mainly enriched in epidermal growth factor receptor binding, neurotrophin binding, and other related molecular functions, as well as in the regulation of platelet activation, monocyte differentiation, and other biological processes, as shown in Figure 4b. To validate the microarray results, we selected ten DE genes that were previously reported to be related to tumors, as shown in Table 2, and verified their expression using RT-qPCR in SK-Hep-1 and HepG2 cells before and after FABP5 knockdown. We found that four genes in both methods (microarray analysis and PCR) showed similar patterns for the selected genes (Figure 4c and Supplementary Figure 2). Of the genes analyzed, KLF9 attracted our attention. It has been reported that KLF9 regulates diverse biological processes, including cell proliferation, differentiation, migration, and apoptosis. It is also closely associated with the occurrence and development of tumors.^20^ In this study, KLF9 was significantly upregulated in the SK-Hep-1 and HepG2 cells upon FABP5 knockdown. We examined KLF9 expression in 48 HCC tissues using RT-qPCR and western blot analysis. KLF9 was downregulated in tumor tissues compared with matched adjacent normal tissues (Figures 4(d,e) and Supplementary Figure 1B). HCC patients with low levels of KLF9 mRNA expression showed low overall survival in TCGA data set (Figures 4f). FABP5 protein expression was negatively correlated with KLF9 expression (r = −0.310, p < .05, Figure 4g). These results suggest that FABP5 may promote HCC development by regulating KLF9 expression.

**Table 2.** Ten genes differentially expressed in the microarray selected and verified their expression by RT-qPCR.

	Gene name
1	NUPR1
2	CCL2
3	CXCL2
4	E2F2
5	FGF12
6	PTPRU
7	KAL1
8	TFPI2
9	AKAP12
10	KLF9

### FABP5 promoted HCC cell proliferation, migration, and invasion by inhibiting KLF9 in vitro

**Figure 5.**
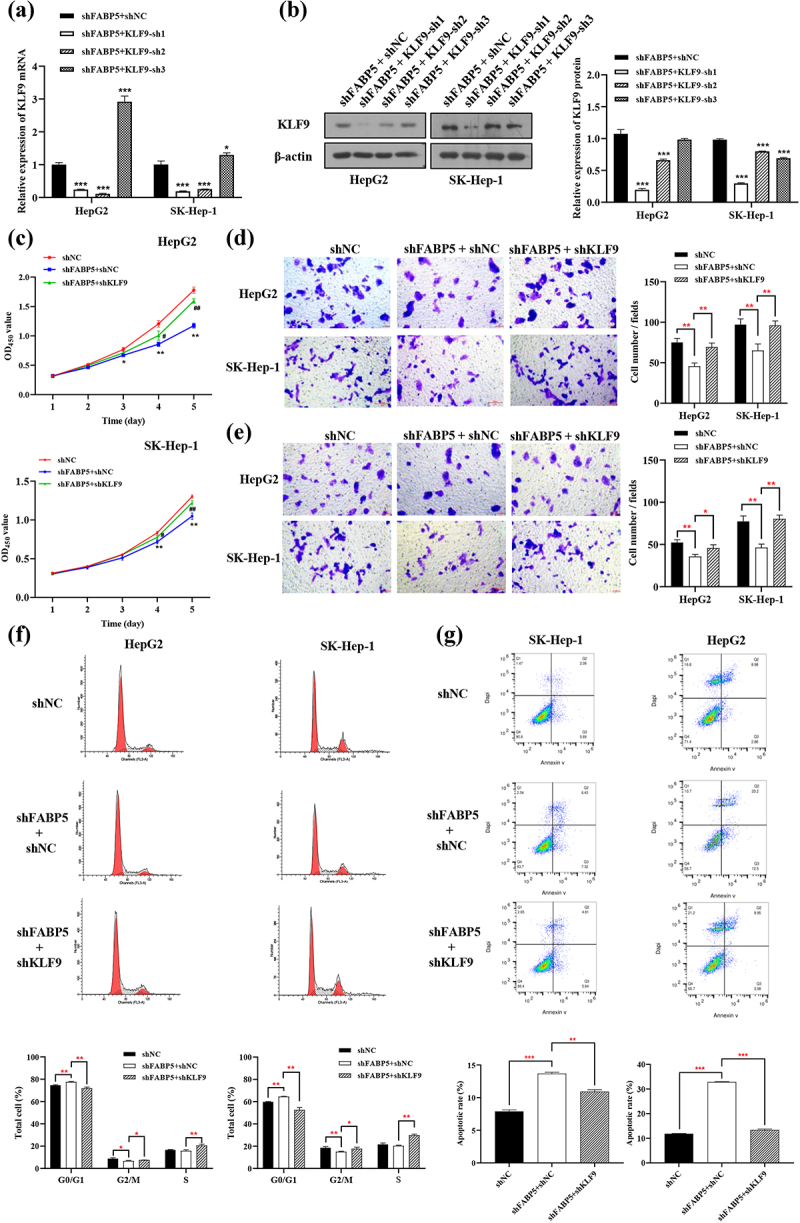
KLF9 knockdown reversed the phenotypes caused by FABP5 knockdown in HCC cells. a-b, HepG2 and SK-Hep-1 cells upon co-transfection of shFABP5 with shKLF9 showed significantly reduced KLF9 expression at the mRNA and protein levels. c, CCK-8 assay showed that shKLF9 significantly reversed the inhibition of proliferation induced by FABP5 knockdown in HCC cells. d, transwell migration assay revealed that shKLF9 significantly rescued the migratory inhibition induced FABP5 knockdown in HCC cells. e, transwell invasion assay revealed that shKLF9 significantly rescued the inhibition of invasion induced by FABP5 knockdown in HCC cells. F, KLF9 knockdown reversed the cell cycle arrest caused by FABP5 knockdown in HCC cells. PI fluorescence pattern was applied for cell-cycle distribution. G, KLF9 knockdown reversed the apoptosis caused by FABP5 knockdown in HCC cells. HepG2 and SK-Hep-1 cells were stained with Annexin-V FITC and PI to be measured apoptosis. Apoptotic cells are presented in the right-lower (Q3, early apoptosis) and right-upper (Q2, late apoptosis) quadrants of the plots. Results are presented as mean ± S.E.M. (N = 3). * p < .05, ** p < .01, and ***p < .001 as compared with the vehicle control. Results were averaged from three independent experiments and presented as percentage of control levels. FITC, fluorescein isothiocyanate; PI, propidium iodide.

We hypothesized that KLF9 may mediate the function of FABP5 in suppressing HCC cell proliferation and metastasis. To test this hypothesis, rescue assays were conducted by co-transfection with shFABP5 with shKLF9 in SK-Hep-1 and HepG2 cells. We selected three KLF9 targets for the knockout experiment. Compared with the shFABP5 group, SK-Hep-1 and HepG2 cells co-transfected with shFABP5 and KLF9-sh1 showed significantly reduced KLF9 expression levels Figures 5(a,b). Thus, shFABP5 with KLF9-sh1 was selected for subsequent experiments. As shown in Figure 5c, suppression of KLF9 rescued the inhibition of cell proliferation induced by FABP5 knockdown. Transwell assays showed that attenuation of KLF9 reversed the effects of FABP5 silencing on the migration of HCC cells (Figure 5d). The invasion assay revealed that shKLF9 significantly rescued the inhibition of invasion induced by FABP5 silencing in HCC cells (Figure 5e). In addition, inhibition of KLF9 expression reversed cell cycle arrest and apoptosis induced by FABP5 knockdown. Figures 5(f,g). These results demonstrated that FABP5 promotes HCC progression by suppressing the expression of KLF9 *in vitro*.

### FABP5 suppresses KLF9 to potentiate PI3K/AKT signaling

**Figure 6.**
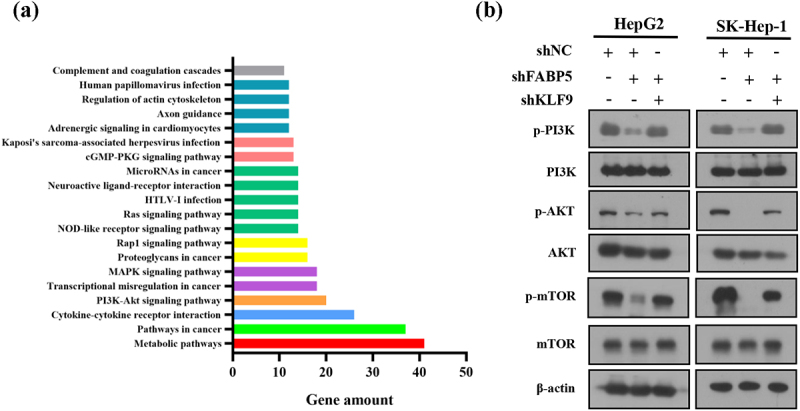
PI3K/AKT signaling pathway was involved in FABP5-induced KLF9 downregulation. a, KEGG pathway enrichment analysis of top 20 differentially expressed signaling pathways in FABP5 knockdown group compared with control group in SK-Hep-1 cells. b, PI3K/AKT signaling pathway was analyzed by western blotting in the indicated groups.

Kyoto Encyclopedia of Genes and Genomes (KEGG) pathway enrichment analysis of DE genes revealed that PI3K/AKT was one of the top twenty differentially expressed signaling pathways (Figure 6a), suggesting that the PI3K/AKT signaling pathway may be involved in FABP5-induced cell proliferation and migration. To examine the potential involvement of PI3K/AKT signaling in FABP5-induced KLF9 downregulation, the phosphorylation levels of PI3K, AKT, and mTOR were measured by western blot analysis in stable HepG2 shFABP5 and SK-Hep1 shFABP5 clones. We found that phosphorylation of PI3K, AKT, and mTOR was decreased in FABP5-knockdown HCC cells and could be reversed by KLF9 gene silencing (Figure 6b and Supplementary Figure 3). Taken together, these data suggest that reduced FABP5 expression inhibits KLF9 expression and the PI3K/AKT signaling pathway.

### miR-889-5p influences the regulatory effect of FABP5 on KLF9

**Figure 7.**
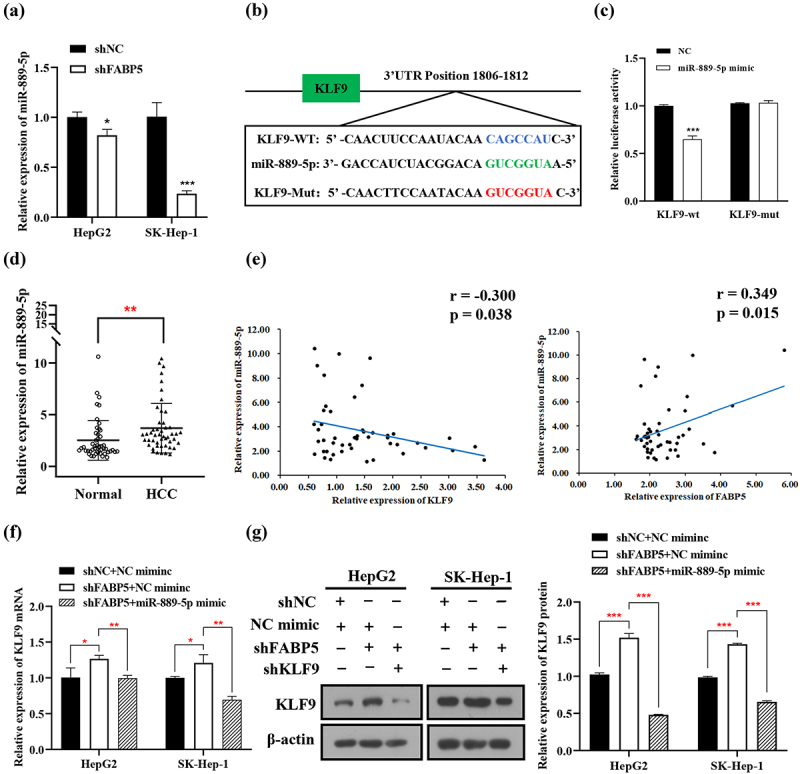
miR-889-5p suppressed KLF9 expression by directly targeted KLF9. a, the expression of miR-889-5p was significantly decreased by FABP5 knockdown in HCC cells. b, bioinformatics analysis predicted that the 3ʹUTR sequence of KLF9 is complementary to the seed sequence of miR-889-5p.The core binding sequences of KLF9 were mutated (in red text). c, dual-luciferase reporter assay was performed to verify that miR-889-5p directly bound to the 3’-UTR sequences of KLF9 in the cells co-transfected with miR-889 mimics or NC with pGL3-KLF9-WT or pGL3-KLF9-Mut. d, relative expression of miR-889-5p in 48 HCC tissues compared with adjacent normal tissues was detected by RT-qPCR. E, miR-889-5p expression was negatively correlated with KLF9 expression and positively correlated with FABP5 expression in HCC tissues. f-g, increased expression of miR-889-5p attenuated KLF9 mRNA and protein expression of in HCC cells with shFABP5 treatment. *p < .05, **p < .01, and ***p < .001 as compared with the vehicle control.

To investigate the specific mechanism of FABP5-regulated KLF9 function, we analyzed the potential upstream target miRNA of KLF9 using targetscan v7.1 and miRDB databases. We focused on ten candidate miRNAs (as shown in Table 3) related to HCC prognosis in the TCGA database. miR-889-5p, miR-26a-5p, miR-636, let-7d-3p, and miR-106b-5p were significantly decreased in the FABP5-knockdown HepG2 and SK-Hep-1 cells (Figure 7a and Supplementary Figure 4). Compared with other potential miRNAs, the expression level of miR-889-5p was the lowest in the FABP5-knockdown HCC cells. Therefore, miR-889-5p was selected for further study. To investigate the relationship between miR-889-5p and FABP5, a dual luciferase assay was conducted in the 293 T cells. miR-889-5p was predicted to have a binding site at the 3-UTR of KLF9. The KLF9 mutation site is illustrated in Figure 7b. Luciferase activity was significantly decreased by treatment with pGL3-KLF9-wt and miR-889-5p mimics. However, the luciferase activity in cells transfected with pGL3-KLF9-mut showed no significant difference following treatment with the miR-899-5p mimic (Figure 7c). These results indicate that miR‑889-5p targets the 3’‑UTR of KLF9 mRNA directly. Next, we compared the miR-889-5p expression in HCC tissues to that in adjacent normal tissues. RT-qPCR analysis showed that miR-889-5p was significantly upregulated in tumor tissues (Figures 7d), while miR-889-5p expression was negatively associated with KLF9 expression in HCC tissues and positively associated with FABP5 expression in HCC tissues (Figures 7e). Next, the effect of miR-889-5p on FABP5-regulated KLF9 expression was further examined. The expression of KLF9 was increased by treatment with shFABP5; however, after combined treatment with the miR-889-5p mimic and shFABP5, the expression of KLF9 was significantly lower compared with the shFABP5 treatment group Figures 7(f,g). Overall, miR-889-5p may be associated with FABP5-regulated KLF9 expression.

**Table 3.** Ten candidate miRNAs target KLF9 through targetscan v7.1 and miRDB databases.

	Gene name
1	Hsa-miR-26a-5p
2	Hsa-miR-889-5p
3	Hsa-miR-889-3p
4	Hsa-miR-636
5	Hsa-Let-7d-3p
6	Hsa-miR-378
7	Hsa-miR-30d
8	Hsa-miR-660-5p
9	Hsa-miR-21
10	Hsa-miR-106b-5

### FABP5 inhibits the expression of miR-889-5p by regulating CREB protein phosphorylation

**Figure 8.**
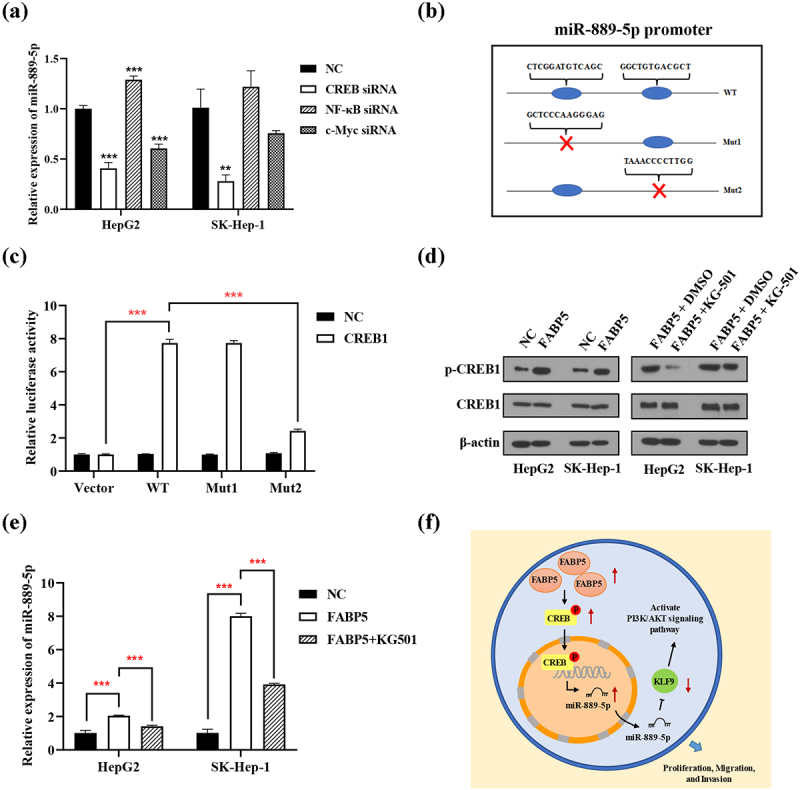
FABP5 inhibits the expression of miR-889-5p by regulating CREB protein phosphorylation. a, the siRNA of NF-κB, c-Myc and CREB were transiently transferred into HepG2 and SK-Hep-1 cells. The expression of miR-889-5p was detected by RT-qPCR. b, the designed mutative model of miR-889-5p promoter. c, a luciferase reporter promoter system was employed to confirm CREB bind to miR-889-5p promoter. d, the expression of total and phosphorylated CREB protein were detected by western blotting in the indicated groups. e, CREB inhibitor KG-501 reverse the effect of FABP5 overexpression on miR-889-5p expression in HCC cells. **p < .01 and ***p < .001 as compared with the vehicle control. e, the scheme of the mechanism by which FABP5 affects HCC tumorigenesis. FABP5 improves CREB protein phosphorylation to upregulate the miR-889-5p expression by CREB binding to the miR-889-5p promoter region, whereby leading to downregulation of KLF9 by miR-889-5p binding to the 3ʹ-UTR of the KLF9 mRNA, potentiating the PI3K/AKT signaling pathway and promoting the proliferation, migration, and invasion of HCC cells.

Both PROMO and JASPAR database were used to predict the transcription factors of miR-889-5p. Based on bioinformatics analysis and previous literatures, the transcription factors NF-κB, c-Myc, and CREB were selected candidates for transcriptional regulation of miR-889-5p promoter. NF-κB, c-Myc, and CREB siRNA were transiently transfected into HepG2 and SK-Hep-1 cells. The expression of miR-889-5p was detected using RT-qPCR. CREB knockdown reduced the expression of miR-889-5p in SK-Hep-1 and HepG2 cells. CREB, but not NF-κB and c-Myc, positively regulated the expression of miR-889-5p (Figure 8a). A luciferase reporter promoter system was used to confirm that CREB was binding to the miR-889-5p promoter. Wild-type (WT) or mutated miR-889-5p promoters (Mut-1 and Mut-2) were cloned into a luciferase reporter vector. The miR-889-5p mutation sites are illustrated in Figure 8b. Ectopic CREB expression markedly improved the luciferase activity of the WT and Mut-1 miR-889-5p promoters but not the Mut-2 promoter, implying that Mut-2 may be the primary binding positions of the CREB and miR-889-5p promoters (Figure 8c). Moreover, overexpression of FABP5 significantly increased the expression of phosphorylated CREB (Figure 8d and Supplementary Figure 5). To investigate whether FABP5 upregulates miR-889-5p via CREB, the CREB inhibitor KG-501 was used. In the HCC cells overexpressing FABP5 treated with the CREB inhibitor KG-501, CREB phosphorylation were significantly decreased compared with the untreated FABP5 overexpression group (Figure 8d). The CREB inhibitor KG-501 reversed the effect of FABP5 overexpression on miR-889-5p expression (Figure 8e). Based on this evidence, we suggest that FABP5 promotes CREB phosphorylation and that this is a result of improved miR-889-5p transcriptional activity.

## Discussion

In this study, we confirmed that FABP5 plays a role in HCC. We demonstrated that FABP5 is highly expressed in human HCC tissues, which confirms the findings of previous studies.^21^ Moreover, we found that FABP5 was involved in HCC cell proliferation, migration, and invasion, which is in agreement with a previous study.^22^ The specific mechanisms underlying the upregulation of FABP5 in various human tumors have also been discussed for other cancers. Overexpression of FABP5 in prostate cancer cells can be attributed to hypomethylation of the CpG island in its promoter region, along with upregulation of the direct trans-acting factors SP1 and c-MYC.^9^ Hypoxia downregulated the expression of miR-144-3p, which subsequently increased the expression of FABP5 in cervical cancer.^13^ The different regulatory networks and molecular mechanisms of FABP5 in numerous other cancers still require further study. In this study, we focused on the mechanism by which FABP5 influences HCC progression. Mechanically and most importantly, we found that the effects of FABP5 appear to work through the upregulation of phosphorylated CREB binding to the miR-889-5p promoter, which in turn leads to upregulation of miR-889-5p. Increased expression of miR-889-5p seems to contribute to the downregulation of KLF9, which in turn inhibits the PI3K-AKT signaling pathway. Notably, we made the novel discovery that FABP5 influences the progression of HCC via the CREB/miR-889-5p/KLF9/PI3K-AKT signaling pathway.

CREB belongs to the leucine zipper class of transcription factors and participates in numerous cellular processes, including proliferation, apoptosis, and autophagy.^23^ Transcriptional regulation depends on CREB phosphorylation status.^24^ Phosphorylated CREB enters the nucleus and binds to specific sites of cAMP response element (CRE) as a transcription factor, thus regulating the expression of target genes.^25^ Accumulating evidence has established the proto-oncogenic role of CREB in tumorigenesis in many different types of malignancies. Previous studies have indicated that CREB was ubiquitously overexpressed in almost all malignant tumors and the overexpression of CREB could dramatically promote tumor proliferation and tumorigenesis in breast cancer, lung cancer, and liver cancer.^26–28^ Moreover, the expression levels of CREB in HCC have been shown to be negatively correlated with prognosis.^29^ In recent years, it has been found that CREB in human tumors regulates the expression of multiple miRNAs, such as miR-9 in glioma and miR-373 in pancreatic cancer.^30,31^ Interestingly, we found strong evidence that FABP5 regulates CREB phosphorylation. Further *in vitro* experiments proved that the upregulation of FABP5 in HCC could increase the expression of miR-889-5p via CREB, which enhances the promoter activity of miR-889-5p. But whether FABP5 is the direct kinase of CREB and how FABP5 regulates CREB phosphorylation have not been determined, which needs further research.

Previous studies have demonstrated that miR-889 is dysregulated in various cancers, such as colon cancer, osteosarcoma, and esophageal cancer. miR-889 promotes colon cancer cell proliferation by inhibiting DAB2IP transcription.^32^ miR-889 can inhibit the expression of myeloid cell nuclear differentiation antigen (MNDA) targets cell cycle regulation in order to influence the progression of osteosarcoma.^33^ However, miR-889 has been shown to have the opposite effect on non-small cell lung cancers (NSCLCs). miR-889 expression levels were found to be significantly reduced in NSCLC, and miR-889 overexpression suppressed the proliferation and invasiveness of NSCLC cells.^34^ These observations suggest that miR-889 may play different roles in different cancers. However, its expression in liver cancer is not well understood. In our study, we found that miR-889-5p expression was significantly upregulated in HCC tissues.

Based on gene chip analyses, we identified KLF9 as a downstream gene target of FABP5. Krüppel-like factors (KLF) is a family of transcription factors with three highly conserved C2H2 zinc finger structures at the carboxyl end, which is widely found in various tissues and has been shown to control essential cellular processes such as proliferation, apoptosis, migration, and differentiation.^35,36^ KLF9, one of the most important members of the KLF family, regulates gene expression by activating or inhibiting promoters with rich GC boxes.^37^ Aberrant expression of KLF9 leads to cellular disorders and influences the occurrence and development of various diseases, including cancer. Emerging studies have demonstrated that KLF9 is commonly downregulated and serves as a tumor suppressor in multiple malignancies, which is related to the poor prognosis of cancer patients with downregulated KLF9 expression.^38–40^ Fu et al.^41^ reported that KLF9 mRNA and protein levels were decreased in HCC tissues compared with normal liver tissues and that the upregulation of KLF9 has anti-proliferative and pro-apoptotic properties in HepG2 cells. Sun et al.^42^ obtained similar results and found that by binding to the p53 promoter, KLF9 upregulates p53 levels, and KLF9 overexpression significantly promotes tumor regression in xenograft models. These results suggest an antitumor role for KLF9 in HCC. In the present study, we demonstrated that FABP5 promoted the proliferation and migration of HCC cells by inhibiting KLF9 expression. KLF9 was significantly downregulated in HCC tissues and was negatively associated with FABP5 expression.

KLF9 is regulated by various miRNAs. Huang et al.^43^ reported that miR-140-5p significantly suppressed the expression of KLF9 by binding to the 3ʹ-UTR of KLF9 mRNA, which promoted the progression of renal cell carcinoma. He et al.^44^ reported that miR-636 promotes the proliferation of bladder cancer cells by decreasing the expression of KLF9 upon binding to the 3’-UTR of its mRNA. miR-135a-5p and miR-135b-5p are involved in FABP5-regulated KLF9 expression in colorectal cancer.^45^ Interestingly, we found that miR-889-5p can target the 3ʹ-UTR of KLF9 mRNA. miR-889-5p expression was positively correlated with FABP5 expression and negatively correlated with KLF9 expression. In addition, treatment with the miR-889-5p mimic attenuated the increase in KLF9 expression induced by FABP5 knockout. These results suggest that FABP5 inhibits the expression of KLF9 by miR-889-5p, which promotes the proliferation and migration of HCC cells.

The results of transcriptional profiling demonstrated that FABP5 knockdown obviously inhibited the PI3K/AKT signaling pathway. PI3K/AKT pathway activation by extracellular signals has been observed in HCC and contributes to tumorigenesis.^46^ Intriguingly, LV et al. found that FABP5 regulated the proliferation of clear cell renal cell carcinoma via the PI3K/AKT signaling pathway.^47^ In the present study, FABP5 silencing significantly decreased the expression of PI3K, p-AKT, and p-mTOR. We hypothesized that FABP5 may regulate the PI3K/AKT signaling pathway via KLF9. To test this hypothesis, KLF9 was silenced in FABP5 knockdown HCC cells. The results demonstrated that KLF9 silencing reversed the p-PI3K, p-AKT, and p-mTOR expression levels in HCC cells, suggesting that FABP5 regulates the PI3K/AKT signaling pathway by inhibiting KLF9 expression.

## Conclusion

In summary, our study demonstrated that FABP5 promotes the proliferation and migration of HCC through the CREB/miR-889-5p/KLF9 axis and provided novel insights into the pathogenesis of HCC. The schematic representation was as shown in figure 8f. The discovery of this signaling pathway and its critical involvement in the progression of HCC serves as the first step in improving our understanding of HCC and aiding treatment. There are some limitations of the current study. First, the number of clinical samples was relatively small, the results still need to verify by larger number of cohorts. Second, we confirmed that FABP5 affect CREB protein phosphorylation, but the mechanism of regulation has not been studied in this study. Although we revealed the involvement of FABP5, CREB, miR-889-5p and KLF9 in HCC progression, we cannot prove entirely that they constitute a single signaling pathway. Therefore, further experiments could identify and confirm the complete upstream-downstream relationship of these signaling molecules.

## Supplementary Material

Supplemental Material
