# Supplementary material for: Fatty acid binding protein 5 promotes the proliferation, migration, and invasion of hepatocellular carcinoma cells by degradation of Krüppel-like factor 9 mediated by miR-889-5p via cAMP-response element binding protein
Source: Cancer Biol Ther. 2022 Jul 11;23(1):424–38. doi: 10.1080/15384047.2022.2094670 (PMC9275499; doi:10.1080/15384047.2022.2094670)
Supplement: Supplemental Material [file KCBT_A_2094670_SM1257.zip › Downloads.zip!/Supplementary material 6 Ethical Approval.pdf]

涉及人体的生物医学研究审查意见

编号：LW2021086

我院唐艳萍在贵刊发表的论文《Fatty acid binding protein 5 promotes the proliferation, migration, and invasion of hepatocellular carcinoma cells by degradation of Krüppel-like factor 9 mediated by miR-889-5p via cAMP-response element binding protein》的有关研究及所涉及的人类遗传资源材料，经广西医科大学附属肿瘤医院伦理委员会审查，认为该研究符合医学伦理学要求。

广西医科大学附属肿瘤医院伦理委员会

2021 年 10 月 25 日

涉及实验动物伦理的审查意见

编号：LW2021087

我院 唐艳萍 在贵刊发表的论文《Fatty acid binding protein 5 promotes the proliferation, migration, and invasion of hepatocellular carcinoma cells by degradation of Krüppel-like factor 9 mediated by miR-889-5p via cAMP-response element binding protein》的有关研究及所涉及的实验动物有关材料，经广西医科大学附属肿瘤医院伦理委员会审查，认为该研究符合医学伦理学要求。

广西医科大学附属肿瘤医院伦理委员会

2021 年 10 月 25 日
